# Successful Resuscitation in a Model of Asphyxia and Hemorrhage to Test Different Volume Resuscitation Strategies. A Study in Newborn Piglets After Transition

**DOI:** 10.3389/fped.2018.00192

**Published:** 2018-07-10

**Authors:** Marc R. Mendler, Stephan Schwarz, Lisbeth Hechenrieder, Steven Kurth, Birte Weber, Severin Höfler, Miriam Kalbitz, Benjamin Mayer, Helmut D. Hummler

**Affiliations:** ^1^Division of Neonatology and Pediatric Critical Care, Department of Pediatrics and Adolescent Medicine, Ulm University, Ulm, Germany; ^2^Department of Traumatology, Hand, Plastic, and Reconstructive Surgery, Center of Surgery, University of Ulm, Ulm, Germany; ^3^Institute of Epidemiology and Medical Biometry, Ulm University, Ulm, Germany; ^4^Division of Neonatology, Department of Pediatrics, Sidra Medicine, Doha, Qatar

**Keywords:** neonatal hemorrhage, neonatal resuscitation, volume resuscitation, crystalloid solution, blood transfusion, neonatal shock, ROSC

## Abstract

**Background:** Evidence for recommendations on the use of volume expansion during cardiopulmonary resuscitation in newborn infants is limited.

**Objectives:** To develop a newborn piglet model with asphyxia, hemorrhage, and cardiac arrest to test different volume resuscitation on return of spontaneous circulation (ROSC). We hypothesized that immediate red cell transfusion reduces time to ROSC as compared to the use of an isotonic crystalloid fluid.

**Methods:** Forty-four anaesthetized and intubated newborn piglets [age 32 h (12–44 h), weight 1,220 g (1,060–1,495g), Median (IQR)] were exposed to hypoxia and blood loss until asystole occurred. At this point they were randomized into two groups: (1) Crystalloid group: receiving isotonic sodium chloride (*n* = 22). (2) Early transfusion group: receiving blood transfusion (*n* = 22). In all other ways the piglets were resuscitated according to ILCOR 2015 guidelines [including respiratory support, chest compressions (CC) and epinephrine use]. One hour after ROSC piglets from the crystalloid group were randomized in two sub-groups: late blood transfusion and infusion of isotonic sodium chloride to investigate the effects of a late transfusion on hemodynamic parameters.

**Results:** All animals achieved ROSC. Comparing the crystalloid to early blood transfusion group blood loss was 30.7 ml/kg (22.3–39.6 ml/kg) vs. 34.6 ml/kg (25.2–44.7 ml/kg), Median (IQR). Eleven subjects did not receive volume expansion as ROSC occurred rapidly. Thirty-three animals received volume expansion (16 vs. 17 in the crystalloid vs. early transfusion group). 14.1% vs. 10.5% of previously extracted blood volume in the crystalloid vs. early transfusion group was infused before ROSC. There was no significant difference in time to ROSC between groups [crystalloid group: 164 s (129–198 s), early transfusion group: 163 s (162–199 s), Median (IQR)] with no difference in epinephrine use.

**Conclusions:** Early blood transfusion compared to crystalloid did not reduce time to ROSC, although our model included only a moderate degree of hemorrhage and ROSC occurred early in 11 subjects before any volume resuscitation occurred.

## Introduction

Although adequate ventilation is the key for an effective resuscitation in the delivery room, some newborns require further assistance such as chest compressions (CC), epinephrine and in specific cases even volume resuscitation in particular in the context of neonatal shock (1). Impaired placental function resulting in fetal asphyxia as well as hypovolemia secondary to fetal blood loss (feto-maternal transfusion or a premature placental abruption) and sepsis are factors contributing to poor conditions at birth (2, 3). Therefore, ILCOR guidelines recommend volume replacement using blood or isotonic crystalloid solution (4), if the newborn infant does not respond to ventilation and CPR and this may be lifesaving (2). However, there is only limited information available on the efficacy of different types of volume replacement. In addition, O-negative, CMV-negative blood is not readily available in all delivery services but may be arranged if considered necessary. In some of these cases placental blood transfusion may be used, as this may be readily available, however data is limited to few retrospective observations as prospective studies are extremely difficult (2) (Table 1). Therefore, we designed this study to establish a model of asphyxia and hemorrhage. Based on our established model of asphyxia (15) we added a component of standardized hemorrhage to evaluate different volume resuscitation strategies in neonatal piglets. We hypothesized that use of early red cell transfusion reduces time to return of spontaneous circulation (ROSC) as compared to the use of an isotonic crystalloid fluid during resuscitation.

**Table 1:** Selected studies about volume expansion in neonates.

**References**	**Type**	**Design/Objective**	**Population**	**Main finding and/or key messages**
**STUDIES CITED IN GUIDELINES**				
Kirkman et al. (5)	Human, case series	Descriptive cohort study	Ten nearly term neonates	The authors described different aspects of post-hemorrhagic anemia and shock due to placental causes
Wyckoff et al. (6)	Human, retrospective review	Descriptive cohort study	Resuscitation registry, 13 infants >34 weeks receiving volume expansion between 01/99 and 06/01	Of 37,972 infants, 23 received CPR, including 13 with volume expansion. 10 of 13 received volume expansion for persistent bradycardia despite CPR, and only three of 13 received volume expansion for suspicion of hypovolemia with poor perfusion
Wyckoff et al. (7)	Animal, prospective	RCT: To compare albumin, normal saline, and SHAM on development of pulmonary edema and restoration of mean arterial pressure during resuscitation of asphyxiated piglets	37 Yucatan miniature swine, (weight 2.2 ± 0.7 kg, postnatal age: 8 ± 4 d)	Volume expansion during resuscitation did not improve mean arterial pressure, and acute recovery of mean arterial pressure was poorer with normal saline compared with albumin. Volume expansion was associated with increased pulmonary edema. In the absence of hypovolemia, volume expansion during neonatal resuscitation is not beneficial
Mayock et al. (8)	Animal, prospective	RCT: To study cerebral blood flow (CBF) regulation, possibly associated with changes in cerebral venous pressure after acute volume expansion in normovolemic developing animals	Protocol 1: 8 preterm sheep fetuses (weight 0.78 ± 0.14 kg, age 95 ± 3 d) Protocol 2: 8 preterm sheep fetuses (weight 0.73 ± 0.04 kg, age 95 ± 1 d)	Rapid volume expansion in the normoxic normovolemic preterm fetal sheep causes a dilutional anemia without a compensatory increase in CBF and, consequently, a decrease in fetal cerebral oxygen delivery (OD). Severe fetal hypoxemia induced after volume expansion further compromises cerebral OD
**SELECTED STUDIES COMPARING DIFFERENT VOLUME AGENTS**				
Roger et al. (9)	Animal, prospective	RCT: To compare the rapidity of shock reversal with lactated Ringer (LR) or hydroxyethyl starch (HES) at the early phase of controlled haemorrhagic shock	36 piglets: (weight 20–31 kg)	Restoration of mean arterial pressure (MAP) was four times faster with HES than with LR in the early phase of controlled hemorrhagic shock. Time to restore the baseline MAP value of +10% was significantly lower in the HES group. The initial infused volume was 279 ± 119 ml in the HES group and 1,011 ± 561 ml in the LR group
Mauch et al. (10)	Animal, prospective	RCT: To evaluate the effect of a single fast intravenous crystalloid or colloid fluid bolus on blood coagulation as measured by rotation thromboelastometry.	32 piglets (weight 5.1 ± 0.4 kg, age 2–5 weeks)	After moderate but very fast volume loading, HES, and gelatine impair blood coagulation to a larger extent as compared with albumin or normal saline, while no significant differences were observed between both artificial colloids
So et al. (11)	Human, prospective	RCT: To compare the efficacy of a colloid (5% albumin) and a crystalloid (isotonic saline) solution for treating hypotension in mechanically ventilated preterm infants within the first 2 h of life	63 preterm infants (weight 0.54–1.95 kg, gestational age 23–34 weeks)	There was no difference in the volume of the test solutions required between the two groups. Isotonic saline is as effective as 5% albumin for treating hypotension in preterm infants, and it has the additional advantage of causing less fluid retention in the first 48 h
Oca et al. (12)	Human, prospective	RCT: To assess the comparative efficacy of normal saline (NS) and 5% albumin (ALB) for treatment of hypotension in the acutely ill newborn	41 newborn infants < 24 h old (weight 0.62–4.28 kg gestational age 25–40 weeks)	NS was shown to be as effective as ALB for the correction of acute hypotension in the newborn infant
Lynch et al. (13)	Human, prospective	RCT: To compare responses to bolus infusion of 5% albumin (ALB) or normal saline (NS) for hypotension in neonates	41 newborn infants < 24 h old (ALB: weight 1.617 ± 0.838 kg gestational age 30.8 ± 4.4 weeks) (NS: weight 1.528 ± 0.830 kg gestational age 30.1 ± 4.1 weeks)	In hypotensive neonates, ALB results in a greater likelihood of achieving normotension and decreased subsequent use of vasopressors when compared to NS
Shalish et al. (14)	Human, review	To investigate the use and evidence of albumin during resuscitation and in the neonatal intensive care unit		There is currently no clinical evidence or physiological rationale to favor the use of albumin over crystalloids in the delivery room resuscitation. Furthermore, only the study from Wyckoff MH, et al. (Use of Volume Expansion During Delivery Room Resuscitation in Near-Term and Term Infants.) is referenced to this topic.
Finn et al. (2)	Human, review	To review the available literature and current guidelines to determine which infants will benefit from Volume Resuscitation (VR), the frequency of VR, and the choice of agents used.		In a setting with presumed or obvious blood loss such as placental abruption or fetal-to-maternal transfusion, VR therapy may play indeed an important role. However, for other clinical scenarios such as asphyxia, the current set of clinical and technical tools makes it difficult to differentiate the haemodynamically compromised infant who will benefit from volume therapy from the normovolaemic asphyxiated infant who may, potentially, be further compromised by volume therapy. …fresh whole blood should be used if available.

## Methods

### Animal preparation

This experiment was approved by the governmental animal care committee (Regierungspraesidium Tuebingen, Permit No.1262). Forty-four newborn piglets [age: 32 (12–44) h; weighing: 1,220 (1,060–1,495) g; Median (IQR)] were anesthetized with propofol/fentanyl, intubated with uncuffed ET tubes and mechanically ventilated (FiO_2_ = 0.3; PIP = 15 cmH_2_O; PEEP = 5 cmH_2_O; inspiratory time = 0.4 s.; initial ventilator rate = 20/min which was adjusted thereafter to achieve a target PaCO_2_ of 35–45 mmHg) using a newborn-ventilator (Stephanie, Stephan GmbH, Gackenbach, Germany). Rectal temperature was maintained at 39.0–39.5°C using a heating mattress and an overhead warmer. A femoral double-lumen arterial line was placed to measure continuous blood pressure and to obtain routinely arterial blood gas analyses (ABG) and for blood sampling. Blood gases were analyzed using a blood gas analyzer (ABL 800, Radiometer, Willich, Germany) and special heparinized syringes. A femoral double-lumen venous line was placed to measure central venous pressure and for administration of i.v. drugs, maintenance fluids, and for administering volume. Airway, esophageal, and vascular pressures were measured continuously using a standard monitoring system (MP 50, Philips, Hamburg, Germany) and a custom-made data acquisition system (ixTrend, ixellence GmbH, Wildau, Germany).

### Resuscitation procedure

**Figure 1:**
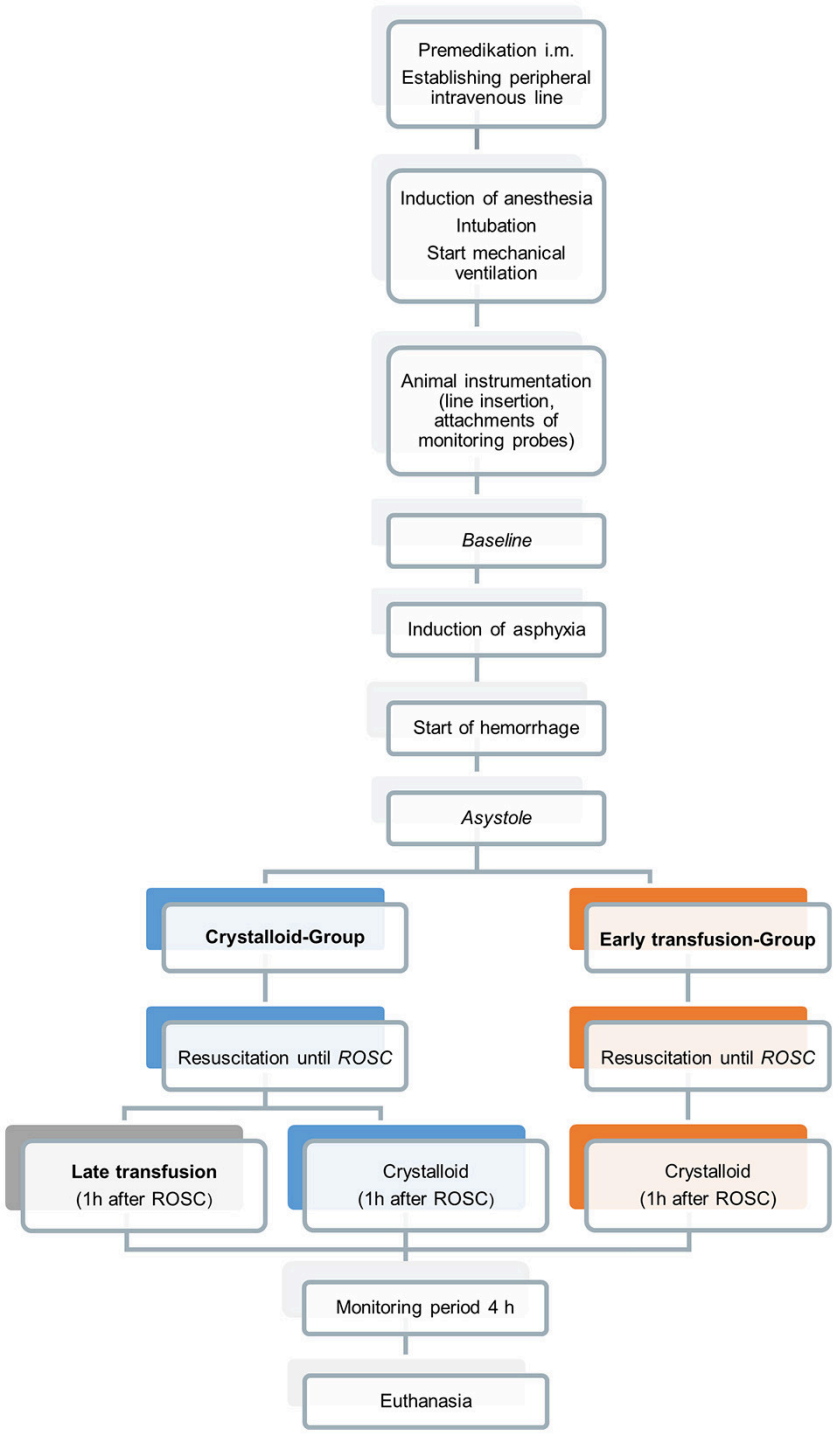
Experimental protocol.

After instrumentation, FiO_2_ was decreased to 0.21 and after 15 min baseline measurements were recorded, and an ABG/blood sample was taken. Thereafter, progressive hypoxia was induced by reducing FiO_2_ to 0.08, adding CO_2_ (FiCO_2_ = 0.07) and by reducing ventilator rate by 10/min every 10 min. At 12 min or once a pH < 7.0 was achieved (whichever occurred first), hypovolemia was induced by continuous and standardized removal of blood (2 ml/kg/min) from the arterial line, using a negative pressure laboratory pump (Syringe pump LA-160, Landgraf Laborsysteme, Langenhagen, Germany). This approach was used to limit variability in the degree of hemorrhage and asphyxia components and the time limit was based on data from of our previous study (15). Blood volume depletion was continued until cardiac arrest occurred. The extracted blood was anticoagulated using a standard anticoagulant mixture and stored in perfusion syringes to be used eventually for re-transfusion. Asystole was defined as loss of pulsatility in the arterial blood pressure waveform along with loss of regular ECG activity. At this time blood extraction was stopped, an ABG was taken, respiratory support was discontinued for 30 s [mimicking the period of initial steps of the Neonatal Resuscitation Program algorithm (4)] and restarted thereafter. Animals were then randomized into one of the following two groups (Figure 1):

1. Crystalloid group: receiving sodium chloride solution 0.9%, and
2. Early transfusion group: receiving the animal's own anticoagulated blood

During resuscitation both groups received mechanical ventilation (PIP/PEEP = 25/5 cmH_2_O, *f* = 30/min, *T*_insp_ = 0.4 s). According to current guidelines a FiO_2_ = 0.21 was used initially. After 30 s of adequate respiratory support CC were applied with a rate of 90/min with inflations interposed to CC using a 3:1 ratio and the 2-thumb-technique (16). Resuscitation was implemented by a trained 5-member team and CC were always given by the same team member. The appropriate rate of CC was guided using a metronome and CC depth was goal-directed to generate a systolic arterial blood pressure of 50 mmHg. Ninety seconds after asystole, FiO_2_ was increased to 1.0 and epinephrine (20 μg/kg) was given via the central venous line and repeated every 3 min. When resuscitation was required to be continued 120 s after asystole, volume expansion was started at a rate of 10 ml/kg over 2 min, with the volume choice dependent on the group allocation. A maximum of 3 × 10 ml/kg volume boluses were infused immediately after each other until ROSC occurred, but the infused total volume never exceeded the total volume previously extracted. Resuscitative efforts were continued until a heart rate >60/min was detected, as measured by ECG and a visible arterial blood pressure, which was defined as ROSC in our model. ABG were taken every 2 min during CPR and immediately after ROSC.

A 4-h monitoring period followed ROSC. In case of arterial hypotension [mean arterial pressure (MAP) < 50 mmHg] a single bolus of normal saline (10 ml/kg over 15 min) was given i.v. If MAP did not increase after the bolus, a second bolus of normal saline (10 ml/kg over 15 min) was given and a continuous infusion of Dopamine was started with the dose adjusted according to the response (max. 15 μg/kg/min).

Furthermore, piglets from the crystalloid group were randomized 1 h after ROSC in two sub-groups to investigate the effects of a late transfusion on hemodynamic parameters. One sub-group received late blood transfusion (10 ml/kg over 15 min) and the other sub-group received an infusion of normal saline (10 ml/kg over 15 min). The animals in the early blood-group received an infusion of normal saline at the same time in order to establish comparability regarding volume status. Four hours after ROSC piglets were euthanized using an intravenous overdose of potassium chloride during ongoing anesthesia with subsequent autopsy, where structural and immunological alterations of the heart were analyzed to be reported in another research publication.

### Sample size and data analysis

Based on results of a previous study done by our group, the study was powered to detect a difference in time to ROSC of 90 s (SD 102 s) between the groups with a power of 80% and a two-sided type 1 error of 0.05 (15). The calculated number of animals was 22 per group. Mean (standard deviation) or median (interquartile range) values are given as appropriate. For comparisons of crystalloid and early transfusion group, continuous data were analyzed using a non-parametric Mann-Whitney-U test and categorical variables using a chi-square or Fisher's exact test. For comparison of variables across time beginning 1 h after ROSC continuous data were analyzed using a non-parametric Kruskal-Wallis test. Statistical significance was accepted with a *p* < 0.05, and all secondary variables were tested in an explorative manner.

## Results

### Baseline characteristics

There were no significant differences between the groups at baseline for demographic or outcome variables, except for blood glucose measurements. Blood glucose was slightly but significantly lower in the crystalloid group (Table 2).

**Table 2:** Baseline characteristics.

		**Early transfusion (*n* = 22)**		**Crystalloid (*n* = 22)**	
Age	(h)	32.5	(15.5–52.3)	16.5	(10.5–43.3)
Weight	(g)	1,167	(1,005–1,374)	1,293	(1,060–1,505)
Sex	(male/female)	8/14		11/11	
ctHb	(g/dl)	6.8	(5.6–8.5)	7.6	(7.0–8.6)
Hct	(%)	21.4	(17.7–26.5)	23.8	(21.8–26.7)
SaO_2_	(%)	95.2	(90.9–96.2)	94.1	(90.1–96.0)
PaCO_2_	(mm Hg)	40.4	(38.6–42.3)	40.1	(38.9–41.9)
PaO_2_	(mm Hg)	71.2	(66.9–79.1)	69.3	(61.1–77.0)
pH		7.45	(7.43–7.48)	7.45	(7.42–7.48)
BE	(mmol/l)	4.0	(1.9–5.8)	3.2	(1.5–5.2)
Glucose	(mg/dl)	127	(108–140)	110^*^	(100–123)
Lactate	(mmol/l)	2.1	(1.7–2.4)	2.3	(1.6–2.6)
K^+^	(mmol/l)	4.1	(3.5–4.4)	4.1	(3.8–4.4)
Na^+^	(mmol/l)	137	(133–139)	136	(134–139)
Ca^2+^	(mmol/l)	1.31	(1.28–1.35)	1.31	(1.28–1.35)
HR	(Beats/min)	162	(143–200)	150	(134–179)
MeanBP	(mmHg)	64.9	(56.5–80.0)	65.1	(53.8–75.5)
Paw	(cm H_2_O)	4.9	(4.8–5.0)	4.8^a^	(4.7–4.9)
Pe	(cm H_2_O)	4.1	(3.0–6.8)	5.3^a^	(4.1–5.7)
CVP	(mm Hg)	4.9	(4.4–6.1)	5.0	(4.4 −5.5)
Temp.	(°C)	39.3	(39.2–39.4)	39.3	(39.2–39.4)
PIP	(cm H_2_O)	14	(13–16)	14	(13.8–15)
Vt	(ml/kg)	7.5	(6.5–10.2)	7.4	(6.2–9.2)

*Values are presented as median (IQR); HR, heart rate; BP, blood pressure; Paw, mean airway pressure; Pe, mean esophagus pressure; CVP, central venous pressure; PIP, inspiratory pressure; Vt, tidal volume*.

* *p = 0.004*.

a *n = 21*.

### Characteristics of cardiac arrest

Time of hypoxia and induction of hemorrhage to achieve asystole was similar in both groups. Average blood loss at this time was 30.7 (22.3–39.6) ml/kg in the crystalloid and 34.6 (25.2–44.7) ml/kg in the early transfusion group; Median (IQR). There was no significant difference in any of the variables between the two groups at this time (Table 3).

**Table 3:** Characteristics at asystole.

		**Early transfusion (*****n*** = **22)**		**Crystalloid (*****n*** = **22)**	
Time to asystole	(s)	2,090	(1,820–2,446)	2,167	(1,806–2,418)
Extracted blood	(ml)	43.5	(34.8–51.3)	40	(40.0–47.3)
Extracted blood/kg	(ml/kg)	34.6	(25.2–44.7)	30.7	(22.3–39.6)
ctHb	(g/dl)	5.2	(4.2–7.0)	6.1	(5.1–6.6)
Hct	(%)	16.5	(13.6–21.8)	19.3	(16.0–20.8)
SaO_2_	(%)	11.2	(8.4–14.0)	11.3	(7.6–13.5)
PaCO_2_	(mm Hg)	127	(112–138)	139	(117–155)
PaO_2_	(mm Hg)	25.6	(20.8–29.8)	22.0	(18.7–26.9)
pH		6.69	(6.63–6.72)	6.67	(6.63–6.73)
BE	(mmol/l)	−19.6	(−23.3 to −18.0)	−19.8	(−21.1 to −17.5)
Glucose	(mg/dl)	169	(102–220)	165	(120–204)
Lactate	(mmol/l)	14.1	(12.1–15.3)	12.2	(7.8–15.0)
K^+^	(mmol/l)	6.4	(5.5–6.7)	5.5	(4.8–6.4)
Na^+^	(mmol/l)	142	(138–144)	141	(139–142)
Ca^2+^	(mmol/l)	1.44	(1.41–1.49)	1.44	(1.39–1.50)
MeanBP	(mmHg)	5.3	(8.5–9.6)	6.1	(4.8–6.7)
Paw	(cm H_2_O)	0		0	
Pe	(cm H_2_O)	4.1	(2.7–5.4)	4.1^a^	(2.7–4.1)
CVP	(mmHg)	4.3	(3.3–5.2)	4.6	(3.8–5.8)
Temperature	(°C)	39.3	(39.2–39.4)	39.3	(39.1–39.4)

*Values are presented as median (IQR); BP, blood pressure; Paw, mean airway pressure; Pe, mean esophagus pressure; CVP, central venous pressure*.

a *n = 21*.

### Characteristics of resuscitation

Thirty-three (of the 44) animals received volume expansion, 16 in the crystalloid group and 17 in the early transfusion group (*p* = 0.728), the other 11 animals did not. The amount of volume expansion infused until ROSC occurred was relatively small with 14.1% and 10.5% of extracted blood because ROSC occurred early. The time until ROSC was not significantly different between the two groups. All animals received epinephrine with no significant difference in dosing between groups (Table 4).

**Table 4:** Characteristics immediately after return of spontaneous circulation (ROSC).

		**Early transfusion (*n* = 22)**		**Crystalloid (*n* = 22)**	
Time to ROSC	(s)	163	(162–199)	164	(129–198)
Epinephrine	(No. of animals with 1/3/7 doses)	20/1/1		22/0/0	
ctHb	(g/dl)	4.6	(3.8–6.7)	5.6	(4.3–6.5)
Hct	(%)	14.7	(12.2–20.9)	17.7	(13.9–20.3)
SaO_2_	(%)	99.5	(99.8–100)	100	(99.3–100)
PaCO_2_	(mm Hg)	46.8	(32.6–54.6)	39.5	(35.1–49.1)
PaO_2_	(mm Hg)	172	(148–198.3)	200.5^*^	(174.8–255.5)
pH		7.00	(6.85–7.09)	7.03	(6.93–7.11)
BE	(mmol/l)	−18.9	(−24.3 to −16.6)	−18.7	(−21.3 to −16.5)
Glucose	(mg/dl)	195	(152–257)	199	(150–241)
Lactate	(mmol/l)	14.9	(14.2–17.0)	13.3^**^	(7.0–15.0)
K^+^	(mmol/l)	6.8	(6.0–7.6)	5.9^†^	(5.1–6.9)
Na^+^	(mmol/l)	139	(136–141)	138	(137–141)
Ca^2+^	(mmol/l)	1.34	(1.11–1.41)	1.33	(1.30–1.39)
HR	(Beats/min)	174	(149–224)	198	(180–224)
MeanBP	(mmHg)	37.9	(26.1–49.3)	47.0^‡^	(39.3–58.8)
Paw	(cm H_2_O)	5.0	(4.8–5.1)	4.9^a^	(4.8–5.0)
Pe	(cm H_2_O)	11.0	(8.6–14.4)	9.5^a^	(8.2–14.5)
CVP	(mmHg)	5.7	(4.9–6.8)	5.6	(4.9–7.0)
Temp.	(°C)	39.1	(39.0–39.3)	39.1	(38.9–39.3)
Vt	(ml/kg)	16.1	(9.9–30.4)	19.6	(13.5–25.8)
		**Early transfusion (*****n*** = **17)**		**Crystalloid (*****n*** = **16)**	
Transfused volume	(ml)	4.0	(3.0–8.0)	6.0	(3.6–8.8)
Transfused volume/Extracted blood	(%)	10.5	(6.9–19.6)	14.1	(7.8–25.5)

Values are presented as median (IQR); BP, blood pressure, Paw, mean airway pressure, Pe, mean esophagus pressure, CVP, central venous pressure, Vt, tidal volume;

* *p = 0.007*,

** *p = 0.004*,

† *p = 0.014*,

‡ p = 0.037;

a *n = 21*.

### Return of spontaneous circulation (ROSC)

All animals achieved ROSC. One animal in the crystalloid group and three animals in the early transfusion group died during monitoring period after successful resuscitation (*p* = 0.607).

Median (IQR) time to ROSC was similar with 164 (129–198) s vs. 163 (162–199) s comparing the crystalloid group and the early transfusion group (Table 4).

**Figure 2:**
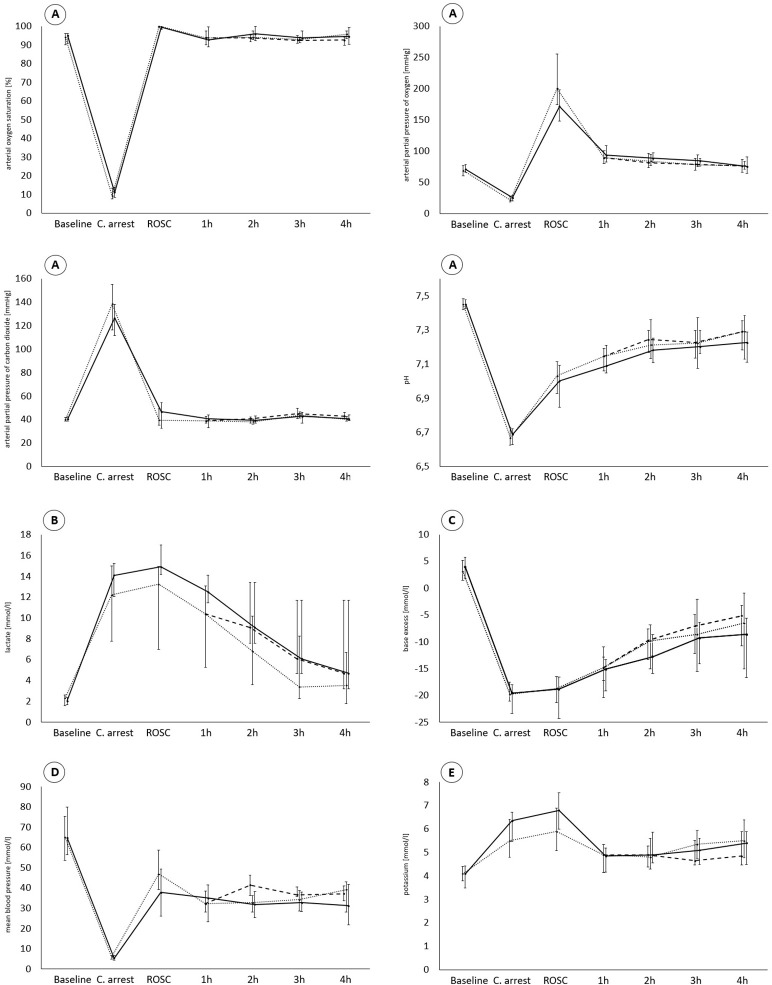
Traces show parameters of gas exchange **(A)**, lactate **(B)**, base excess **(C)**, mean arterial blood pressure **(D)**, and potassium **(E)** during all phases of experiment. ________, early transfusion group; ···············, crystalloid group; – – – – –, late transfusion (in crystalloid group); Values are presented as median (IQR).

Arterial blood gases immediately after ROSC were similar for the two groups, except for partial pressure of oxygen (PaO_2_), potassium, and lactate. Significantly higher levels for potassium and lactate were measured in the early transfusion group and a higher PaO_2_ was observed in the crystalloid group (Table 4, Figure 2). Hemodynamic parameters were similar, except for mean arterial blood pressure, which was significantly higher in the crystalloid group (Table 4).

### Post-resuscitation period

There were no significant differences between the groups regarding blood gases, metabolic, or hemodynamic parameters during the monitoring period (see Supplementary Tables 1–3 for detailed values), except for a significantly higher lactate 1 h after ROSC in the early transfusion group and a significantly higher mean arterial blood pressure 2 h after ROSC in the subgroup (of crystalloid group) receiving late transfusion 1 h after ROSC (Table 5, Figure 2). There were also no significant differences in the use of dopamine or volume boluses supporting circulation (Table 5).

**Table 5:** Significant differences during monitoring period and characteristics at 4 h after ROSC.

**1 h after ROSC**		**Early transf. (*n* = 20)**		**Crystalloid (*n* = 21)**			
**Lactate**	**(mmol/l)**	**12.5**	**(11.5–14.1)**	10.4^*^		(5.3–13.1)	
**2 h after ROSC**		**Early transf. (*n* = 20)**		**Crystalloid with late volume infusion 1 h after ROSC**			
				**Blood (*n* = 10)**		**Sodium chloride 0.9% (*n* = 11)**	
**MeanBP**	**(mmHg)**	**31.9**	**(25.5–38.3)**	41.5^**^	**(36.2–46.4)**	**32.8**	**(28.2–36.3)**
**4 h after ROSC**		**Early transf. (*n* = 19)**		**Crystalloid with late volume infusion 1 h after ROSC**			
				**Blood (*n* = 10)**		**Sodium chloride 0.9% (*n* = 11)**	
ctHb	(g/dl)	4.4	(3.7–6.0)	5.7	(4.9–6.1)	5.2^c^	(4.0–7.2)
Hct	(%)	14	(11.8–18.9)	18.1	(18.7–19.3)	16.3^c^	(13–22.4)
SaO_2_	(%)	94.6	(90.3–99.4)	92.7^b^	(89.9–94.2)	95.8^c^	(93.3–97.6)
PaCO_2_	(mm Hg)	40.6	(39.1–43.8)	43.0	(40.7–46.1)	40.4^c^	(38.6–42.5)
PaO_2_	(mm Hg)	75.6	(65.2–91.2)	76.6	(66.7–87.4)	77.2^c^	(71.3–83.8)
pH		7.23	(7.11–7.29)	7.29	(7.18–7.35)	7.29^c^	(7.13–7.38)
BE	(mmol/l)	−8.6	(−16.7 to −5.6)	−5.2	(−10.7 to −3.2)	−6.5^c^	(−15.0 to −0.9)
Glucose	(mg/dl)	95	(81–122)	99	(84–115)	98^c^	(72–125)
Lactate	(mmol/l)	4.7	(3.2–11.7)	2.7	(2.1–6.1)	3.6^c^	(1.8–6.7)
K^+^	(mmol/l)	5.4	(4.5–5.9)	4.9	(4.5–5.9)	5.5^c^	(4.8–6.4)
Na^+^	(mmol/l)	138	(136–139)	137	(136–138)	137^c^	(134–139)
Ca^2+^	(mmol/l)	1.45	(1.41–1.55)	1.38	(1.35–1.51)	1.41^c^	(1.36–1.54)
HR	(Beats/min)	252	(228–264)	243	(227–258)	222	(192–246)
MeanBP	(mmHg)	31.2^a^	(21.7–41.7)	37.3	(33.8–41.2)	39.2	(28.1–43.0)
Paw	(cm H_2_O)	4.9	(4.8–5.0)	4.9^b^	(4.8–5.1)	4.8	(4.7–4.9)
Pe	(cm H_2_O)	5.4	(4.1–8.2)	5.4^b^	(4.1–7.7)	6.8	(4.1–8.2)
CVP	(mmHg)	4.6	(3.6–6.3)	4.8	(4.1–5.8)	5.5	(4.5–7.6)
Temp.	(°C)	39.2	(39.0–39.4)	39.3	(39.2–39.4)	39.2	(39.1–39.2)
PIP	(cm H_2_O)	16	(13–17)	15	(15–17)	14	(13 −15)
Vt	(ml/kg)	8.2	(6.7–10.2)	9.0^b^	(8.0–10.6)	8.7	(7.1–8.9)
FiO_2_	[%]	23	(21–25)	23^b^	(22–27)	23	(22–25)
Dopamine (cumulative dose)	(mg/kg)	3.2	(2.9–3.9)	3.3	(3.0–3.5)	3.5	(3.1–3.6)

Values are presented as median (IQR); HR, heart rate; BP, blood pressure; Paw, mean airway pressure; Pe, mean esophagus pressure; CVP, central venous pressure; PIP, inspiratory pressure; Vt, tidal volume;

* *p = 0.007*,

** p = 0.006;

a *n = 18*,

b *n = 9*,

c *n = 10*.

## Discussion

Current resuscitation guidelines recommend volume expansion for newborn infants not responding to ventilation and CC, especially if there is a history suggesting fetal blood loss. Guidelines for volume administration were not changed in the current AHA guidelines (2015); therefore, the 2010 recommendations remain in effect (4). Administration of blood or crystalloids with no clear preference for either one is currently recommended (17). Furthermore, ERC guidelines in relation to volume administration were not changed in 2015 (18). Nevertheless, they state that in the absence of suitable blood isotonic crystalloid is the solution of choice. In this guideline four publications are referenced in relation to this topic, including an older case report by Kirkman and Riley from 1959 (5), a retrospective review from Wyckoff et al. (6) and only one randomized study in asphyxiated piglets [comparing use of crystalloids with colloidal infusion showing no difference between groups 7]. A recent review by Finn et al. states that data on different fluids for volume expansion is very limited, because prospective studies remain impracticable and until now only few studies are available on this topic (2).

Crystalloids are readily available, and their costs are low. However, they have a lower intravascular half-life of 30–60 min secondary to immediate loss of fluid into the interstitial space, as well as the lack of oxygen transport capacity as compared to blood. There are also (low) risks for infection (19) and transfusion reactions when blood is used without cross-match (20), as well as for hyperkalemia and increased lactate (21, 22).

Because of paucity of data we used an established model of hypoxic cardiac arrest in newborn piglets (15, 23) and added a standardized protocol for hemorrhage.

Our hypothesis that early blood transfusion reduces time to ROSC during resuscitation compared to using a crystalloid solution was not confirmed. We followed a protocol and were able to reproduce severe asphyxia with very similar degrees of acidosis and hypoxemia in both study groups, confirming that this model is quite stable. We followed closely the published resuscitation guidelines and used epinephrine before volume expansion and all animals received at least one dose of epinephrine, which, because of its positive effect on coronary blood flow, is considered to be important for successful resuscitation (24). Volume depletion was approximately 1/3 of the estimated blood volume. However, it seems that we were able to resuscitate many animals successfully without substantial volume resuscitation, as indicated by the fact that ROSC occurred in 25% of the animals before any volume infusion. It is likely that the degree of hemorrhagic shock was not severe enough to affect successful resuscitation as at ROSC only 12 (7–21) % [Median (IQR)] of depleted blood volume was re-transfused. This is further supported by the fact that time to ROSC is very similar to our previous study (with 150–180 s), which used exactly the same protocol to induce asphyxia, but did not include the hemorrhagic component (15). However, it also indicates that, despite relevant blood loss and asphyxia, resuscitation may be possible in a relevant proportion of subjects with no or only a minor amount of volume replacement, which may be potentially harmful (2), and therefore needs a clear indication.

We observed higher values for potassium and lactate in the early transfusion group after ROSC. This can be explained by the additional acid and potassium load caused by the early transfusion of stored blood in this group. Lactic acidosis may also affect cardiac contractility and thus may explain the lower blood pressure at this time point in the early transfusion group, as described elsewhere (25). The increased lactate was detectable only up to 1 h after ROSC, suggesting that our animals had adequate perfusion and metabolic homeostasis after successful resuscitation. The higher potassium level had already decreased at this time. With a larger transfusion volume, a relevant potassium load needs to be considered along with its side-effects on the heart. Nevertheless, there are clear advantages using blood instead of crystalloids such as a longer intravascular half-life as well as the oxygen transport capacity by red blood cells (26). The higher mean blood pressure observed in the subgroup with the late blood transfusion 1 h after ROSC, may be explained by a more stable blood volume as compared to the subgroup receiving crystalloids. We do not have a clear explanation for the slightly but significantly lower blood glucose in the crystalloid group at the baseline, but we speculate that it may be related to the somewhat younger postnatal age and its related metabolic instability. Glucose supply given by the infusion solution ensured similar conditions during the experiment including the phase of resuscitation.

Our study has several limitations. First, the study was not blinded. However, we applied a standardized protocol for CC and hemodynamic support. Second, we used the animals' whole blood, instead of packed red blood cells, which would contain higher haematocrit and may impose a higher load of potassium due to the longer storage phase. However, in some cases placental blood (with similar hematocrit) may be used as it may be readily available. Third, our animals were anesthetized, which differs from delivery room procedures and may cause interference with ROSC. The second randomization of the animals within the crystalloid group at one hour after ROSC may limit the power to show differences between groups, but only for the secondary outcomes after that time point. Furthermore, our animals had already passed their early phase of postnatal transition. However, despite this limitation, the piglet model is well-established and many groups have used it to study asphyxia and neonatal resuscitation (23). One future direction for research may be cord milking once severe bradycardia or asystole is confirmed, which may induce immediate blood transfusion via the umbilical cord. This should be studied in a transitional model, such as the lamb model of asphyxia arrest. Finally, as mentioned before the degree of volume depletion may not have been severe enough to affect resuscitation significantly and thus our findings may only apply for a mild-moderate degree of hemorrhage.

In summary, we developed our animal model with a hemorrhagic component to assess the influence of different volume resuscitation strategies on ROSC. Our hypothesis that early blood transfusion reduces time to ROSC during resuscitation compared to using a crystalloid solution was not confirmed. However, our model is limited by the fact that only 75% of the animals needed transfusion for ROSC, which occurred already at a time when only 12 (7–21)% [Median (IQR)] of depleted blood volume was re-transfused suggesting that hemorrhagic shock was not severe enough to limit resuscitation success substantially to test our hypothesis. However, we were able to show that, despite severe asphyxia and blood loss, resuscitation procedures were successful with no, or minimal transfusion. Animals required at least one adrenaline dose for successful resuscitation, confirming the central importance of this drug. Further studies are needed to investigate the influence of different volume expansion strategies in a refined model of a more severe degree of hemorrhage to identify a potential threshold of blood loss, where emergency blood transfusion might improve outcome.

## Author contributions

MM and HH conceived and designed the study. MM, SS, LH, SK, BW, and SH performed the animal experiments. MM, LH, SK, MK and BM analyzed the data. MM, BM, and HH interpreted the results obtained. MM drafted the manuscript. MM and HH revised the manuscript. All authors read and approved the final version of the manuscript and agreed to be accountable for all aspects of the work.

### Conflict of interest statement

Fritz Stephan GmbH (Gackenbach, Germany) provided the mechanical ventilator. The authors declare that the research was conducted in the absence of any commercial or financial relationships that could be construed as a potential conflict of interest.
