# Supplementary material for: Successful Resuscitation in a Model of Asphyxia and Hemorrhage to Test Different Volume Resuscitation Strategies. A Study in Newborn Piglets After Transition
Source: Front Pediatr. 2018 Jul 10;6:192. doi: 10.3389/fped.2018.00192 (PMC6048263; doi:10.3389/fped.2018.00192)
Supplement: Supplementary file 1 [file Table_1.pdf]

Supplemental Table 1: Characteristics during monitoring period at **1h after ROSC**: Values are presented as median (IQR); HR = heart rate, BP = blood pressure, Paw = mean airway pressure, Pe = mean esophagus pressure, CVP = central venous pressure, PIP = inspiratory pressure, Vt = tidal volume; * = p=0.007; [a] = n=20

		Early transfusion (n=20)		Crystalloid (n=21)	
ctHb	[g/dl]	4.8	(3.8 – 6.5)	5.6	(4.8 – 5.8)
Hct	[%]	15.2	(12.5 – 20.2)	17.7	(15.2 – 18.4)
SaO_2	[%]	92.8	(89.0 – 99.7)	93.9	(90.1 – 97.6)
$PaCO_2$	[mm Hg]	40.5	(33.0 – 44.1)	38.9	(36.9 – 42.8)
PaO_2	[mm Hg]	93.8	(82.6 – 109.0)	89.8	(80.7 – 101.1)
pH		7.09	(7.05 – 7.21)	7.15	(7.06 – 7.19)
BE	[mmol/l]	-15.1	(-19.1 – -13.3)	-14.8	(-17.2 – -11.0)
Glucose	[mg/dl]	120	(94 – 191)	150	(107 – 167)
Lactate	[mmol/l]	12.5	(11.5 – 14.1)	10.4*	(5.3 – 13.1)
K^+	[mmol/l]	4.9	(4.2 – 5.2)	4.9	(4.2 – 5.4)
Na^+	[mmol/l]	139	(136 – 141)	138	(137 – 140)
Ca^{2+}	[mmol/l]	1.34	(1.28 – 1.40)	1.35	(1.29 – 1.43)
HR	[Beats/min]	255	(235 – 270)	246	(228 – 270)
MeanBP	[mmHg]	35.1	(23.4 – 41.4)	32.3	(28.2 – 38.5)
Paw	[cm H_2O]	4.9	(4.8 – 5.1)	4.8[a]	(4.7 – 5.0)
Pe	[cm H_2O]	6.1	(4.1 – 6.8)	5.4[a]	(4.1 – 6.8)
CVP	[mmHg]	4.0	(3.3 – 4.7)	4.5	(3.6 – 5.3)
Temp.	[°C]	39.3	(39.1 – 39.5)	39.3	(39.2 – 39.4)
PIP	[cm H_2O]	16	(13 – 17)	15	(14 – 16)
Vt	[ml/kg]	9.6	(5.9 – 11.2)	8.9 [a]	(8.3 – 10.7)
FiO_2	[%]	25	(25 – 27)	25 [a]	(25 – 30)
