# Supplementary material for: Successful Resuscitation in a Model of Asphyxia and Hemorrhage to Test Different Volume Resuscitation Strategies. A Study in Newborn Piglets After Transition
Source: Front Pediatr. 2018 Jul 10;6:192. doi: 10.3389/fped.2018.00192 (PMC6048263; doi:10.3389/fped.2018.00192)
Supplement: Supplementary file 2 [file Table_2.pdf]

Supplemental Table 2: Characteristics during monitoring period at **2h after ROSC**: Values are presented as median (IQR); HR = heart rate, BP = blood pressure, Paw = mean airway pressure, Pe = mean esophagus pressure, CVP = central venous pressure, PIP = inspiratory pressure, Vt = tidal volume; * = p=0.006; [a] = n=10, [b] = n=9, [c] = n=8

		Early transf. (n=20)		Crystalloid with late volume infusion 1h after ROSC			
				Blood (n=10)		Sodium chloride 0,9% (n=11)	
ctHb	[g/dl]	4.7	(3.6 – 6.3)	6.1	(5.5 – 6.5)	5.3[a]	(4.2 – 7.3)
Hct	[%]	14.8	(11.8 – 19.8)	19.1	(17.4 – 20.3)	16.8[a]	(13.3 – 22.7)
SaO_2	[%]	96.0	(92.3 – 100)	93.8	(91.9 – 95.4)	94.0[a]	(92.7 – 97.6)
$PaCO_2$	[mm Hg]	39.3	(37.1 – 43.1)	40.9	(37.2 – 42.1)	38.3	(36.1 – 40.5)
PaO_2	[mm Hg]	89.0	(82.1 – 97.8)	82.0	(74.1 – 97.1)	84.3[a]	(77.5 – 95.6)
pH		7.18	(7.11 – 7.25)	7.24	(7.17 – 7.30)	7.21	(7.13 – 7.36)
BE	[mmol/l]	-12.8	(-15.9 – -8.6)	-9.8	(-13.3 – -7.6)	-9.8	(-15.0 – -6.8)
Glucose	[mg/dl]	113	(81 – 151)	149	(85 – 160)	120	(104 - 146)
Lactate	[mmol/l]	9.1	(7.6 – 13.4)	6.6[b]	(5.4 – 10.7)	6.8[a]	(3.6 – 10.2)
K^+	[mmol/l]	4.9	(4.6 – 5.9)	4.9	(4.4 – 5.3)	4.8	(4.3 – 5.6)
Na^+	[mmol/l]	138	(136 – 141)	137	(134 – 140)	137	(136 - 141)
Ca^{2+}	[mmol/l]	1.37	(1.31 – 1.46)	1.28	(1.17 – 1.37)	1.35	(1.29 – 1.41)
HR	[Beats/min]	258	(240 – 275)	255	(228 - 269)	240	(216 – 258)
MeanBP	[mmHg]	31.9	(25.5 – 38.3)	41.5*	(36.2 – 46.4)	32.8	(28.2 – 36.3)
Paw	[cm H_2O]	5.0	(4.7 – 5.0)	4.9[b]	(4.7 – 5.0)	4.8	(4.7 – 4.9)
Pe	[cm H_2O]	6.2	(3.1 – 8.2)	5.0[c]	(4.1 – 6.5)	4.1	(4.1 – 6.8)
CVP	[mmHg]	4.6	(3.4 – 6.5)	4.7	(3.6 – 6.4)	4.0	(3.4 – 6.3)
Temp.	[°C]	39.3	(39.1 – 39.4)	39.3	(39.2 – 39.3)	39.1	(39.1 – 39.3)
PIP	[cm H_2O]	15	(13 – 17)	15	(15 – 16)	14	(13 –15)
Vt	[ml/kg]	9.1	(6.9 – 10.3)	10.1[b]	(8.6 – 11.7)	8.9	(7.7 – 10.4)
FiO_2	[%]	25	(24 - 28)	25[b]	(23 - 27)	24	(23 - 25)
