# Supplementary material for: Successful Resuscitation in a Model of Asphyxia and Hemorrhage to Test Different Volume Resuscitation Strategies. A Study in Newborn Piglets After Transition
Source: Front Pediatr. 2018 Jul 10;6:192. doi: 10.3389/fped.2018.00192 (PMC6048263; doi:10.3389/fped.2018.00192)
Supplement: Supplementary file 3 [file Table_3.pdf]

Supplemental Table 3: Characteristics during monitoring period at **3h after ROSC**: Values are presented as median (IQR); HR = heart rate, BP = mean blood pressure, Paw = mean airway pressure, Pe = mean esophagus pressure, CVP = central venous pressure, PIP = inspiratory pressure, Tv = tidal volume; [a] = n=10, [b] = n=9, [c] = n=8

		Early transf. (n=19)		Crystalloid with late volume infusion 1h after ROSC			
				Blood (n=10)		Sodium chloride 0,9% (n=11)	
ctHb	[g/dl]	4.8	(3.5 – 6.3)	5.9	(5.4 – 6.3)	5.2[a]	(4.3 – 7.1)
Hct	[%]	15.4	(11.3 – 19.8)	18.6	(16.9 – 19.9)	16.5[a]	(13.7 – 22.1)
SaO_2	[%]	93.8	(92.4 – 97.6)	92.5	(90.9 – 95.1)	92.9[b]	(91.2 – 94.3)
$PaCO_2$	[mm Hg]	42.7	(36.9 – 46.1)	45.1	(41.1 – 49.7)	43.6[a]	(41.2 – 46.5)
PaO_2	[mm Hg]	84.8	(76.1 – 89.0)	79.6	(69.9 – 88.9)	78.6[a]	(75.8 – 94.7)
pH		7.20	(7.16 – 7.30)	7.23	(7.14 – 7.30)	7.22[a]	(7.08 – 7.37)
BE	[mmol/l]	-9.3	(-14.1 – -6.3)	-7.1	(-12.2 – -5.0)	-8.6[a]	(-15.5 – -2.1)
Glucose	[mg/dl]	108	(85 – 123)	113	(87 – 132)	103[a]	(94 - 136)
Lactate	[mmol/l]	6.1	(4.7 – 11.7)	4.7	(2.9 – 7.6)	3.4[a]	(2.3 – 8.2)
K^+	[mmol/l]	5.1	(4.5 – 5.6)	4.7	(4.5 – 5.5)	5.4[a]	(4.8 – 5.9)
Na^+	[mmol/l]	138	(135 – 140)	138	(135 – 139)	137[a]	(136 - 139)
Ca^{2+}	[mmol/l]	1.41	(1.36 – 1.54)	1.34	(1.31 – 1.48)	1.41[a]	(1.33 – 1.52)
HR	[Beats/min]	240	(228 – 264)	243	(231 - 266)	234	(204 – 252)
MeanBP	[mmHg]	32.8	(28.5 – 38.1)	36.7	(35.7 – 40.6)	34.3	(28.6 – 38.9)
Paw	[cm H_2O]	4.9	(4.7 – 5.0)	4.8[b]	(4.7 – 5.0)	4.8	(4.7 – 4.8)
Pe	[cm H_2O]	5.4	(4.1 – 8.2)	5.4[c]	(4.1 – 7.5)	5.4	(4.1 – 6.8)
CVP	[mmHg]	4.8	(3.7 – 6.3)	4.9	(4.4 – 6.7)	4.7	(3.9 – 6.3)
Temp.	[°C]	39.3	(39.2 – 39.5)	39.3	(39.2 – 39.4)	39.3	(39.2 – 39.4)
PIP	[cm H_2O]	15	(13 – 17)	15	(15 – 16)	14	(13 –15)
Tv	[ml/kg]	8.5	(6.8 – 10.3)	10.3[b]	(8.6 – 11.0)	8.9	(7.3 – 9.6)
FiO_2	[%]	25	(23 - 26)	25[b]	(23 - 28)	23	(22 - 27)
